# Drama Therapy as a Tool for Peace and Conflict Resolution in Family Dynamics: A Pilot Study

**DOI:** 10.3390/bs15091156

**Published:** 2025-08-25

**Authors:** Lina Haddad Kreidie, Suzanne Wehbe, Sara Sakhi, Karima Anbar, Intisar Al Sabah

**Affiliations:** 1 Communication, Mobility, and Identity Department, School of Arts and Sciences, Lebanese American University, Beirut 11022801, Lebanon; 2 Psychology Department, School of Arts and Sciences, Lebanese American University, Beirut 03797751, Lebanon; suzanewehbeh2000@gmail.com; 3 Social Services Department, Alice Salomon Hochschule, 12627 Berlin, Germany; ssakhi1994@gmail.com; 4 Political Science Department, Ecole Normale Supérieure de Lyon, 69342 Lyon, France; karima.nbr@gmail.com; 5 Dar Al Lulua, Abu Al Hassaniya 24700, Kuwait; intisar@lulua.com

**Keywords:** child maltreatment, Drama Therapy Intervention, family dynamics, mental health

## Abstract

Refugee mothers are at heightened risk of developing negative family dynamics due to traumatic experiences and unstable living conditions, often impacting their children in lasting ways. This partially mixed, explanatory mixed-methods pilot study examines the potential of Drama Therapy as a psychosocial intervention to reduce harmful parenting behaviors and strengthen parent–child relationships. The study engaged 20 refugee mothers who participated in a three-session intervention based on Emunah’s five-phase model. Data collection included pre-intervention demographic information, two standardized psychological scales—The Child–Parent Relationship Scale and the Parent Anger Scale—and post-intervention focus group discussions. The findings indicate that the Drama Therapy Intervention (DTI) helped reduce parental anger and improve emotional regulation, leading to more positive interactions with children and decreased conflict within the family. Focus group insights revealed that the mothers’ ongoing and past traumas significantly shaped their emotional responses and parenting styles. This pilot study highlights the importance of addressing maternal mental health in post-displacement contexts. Although one cannot draw causal inferences of efficacy in the absence of a control group, the findings provide preliminary evidence that Drama Therapy can be an effective tool for reducing parental maltreatment and improving family relationships among refugee populations.

## 1. Introduction

As the world grapples with the highest number of armed conflicts globally since the Second World War, forced displacement and unstable living conditions have reached unprecedented levels (UNHCR, 2023). Naturally, during and after times of conflict, many social issues arise due to instability and the collapse of societal structures. Social cohesion is tested to its limits as communities break down under the stress of war and displacement. Social dynamics shift significantly during crises, as many are deprived of safety and autonomy (Miller & Rasmussen, 2017). Within these complex systems, the family unit is often severely impacted. Parents may lose their ability to keep their children safe, healthy, and within structured systems designed to ensure their well-being and prospects, such as schools. After displacement, many refugee parents must rebuild not only their own lives but also the lives of their children, which have been grossly derailed. These many stressors may inevitably affect parent–child relationships, sometimes leading to maladaptive parenting practices (Simi et al., 2016; Eltanamly et al., 2019). This study explores these complex issues in the context of Lebanon’s refugee population and asks whether psychosocial interventions can mitigate negative effects on mothers.

## 2. Literature Review

### 2.1. What Is Child Maltreatment and What Are Its Implications?

Child maltreatment can be a single or recurring act, or a failure to act, that results in physical, emotional, or psychological harm to a child by their caregiver (National Data Archive on Child Abuse and Neglect, 2007). The World Health Organization (2020) estimates that up to 75% of children worldwide will be subjected to some form of maltreatment between the ages of 2 and 4. According to global prevalence figures, child neglect and physical, sexual, and emotional abuse are widespread. Current statistics show that physical abuse is experienced by 8.0% of children, sexual abuse by 1.6%, neglect by 4.4%, and emotional abuse by 36.3%. These forms of violence and neglect are collectively known as child abuse (Vachon et al., 2015). Such maltreatment has significant psychological consequences for children.

Childhood maltreatment significantly affects a child’s physical and mental well-being, often leaving a lasting impression into adulthood. It can result in a variety of maladaptive behaviors, including smoking, substance abuse, high-risk behavior, and an increased likelihood of revictimization (Kendall-Tackett, 2002). A meta-analysis of different types of child abuse found strong correlations with a wide range of mental health issues, including depression, post-traumatic stress disorder, eating disorders, anxiety, and suicidal behavior (Vachon et al., 2015). Sexual abuse victims are particularly vulnerable to lifelong prevalence of post-traumatic stress disorder, obsessive–compulsive disorder, agoraphobia, social phobia, and sexual disorders compared to the general population (Springer et al., 2003). These mental health issues can also lead to physical health complications, including chronic pain, fatigue, sleep disturbances, headaches, and gastrointestinal issues (Springer et al., 2003).

Victimization at the hands of a caretaker during childhood may lead to lifelong behavioral issues and possible criminal inclinations. While not all victims of childhood abuse develop these issues, evidence suggests a link between early maltreatment and a later likelihood of offending (Local Government Association, 2018). Witnessing domestic violence as a child causes trauma, which can lead to violence in adolescence and adulthood (O’Driscoll, 2017). Physical abuse during childhood may result in violent delinquency in adolescence and early adulthood, along with various social issues, including higher rates of dropping out of high school and being fired from jobs (Lansford et al., 2007). A study found that 23% of former members of violent terrorist groups stated they experienced sexual abuse during childhood or adolescence, while 41% suffered emotional and physical neglect (Simi et al., 2016).

Childhood maltreatment by mothers is a serious and often silent issue affecting millions of children worldwide. Abusive mothers leave lasting scars on the psyches, and sometimes bodies, of their children, often leading to psychological and behavioral complications as they grow up, including the potential to replicate similar behavior when they become parents (Gama et al., 2021). Much of the modern literature focuses primarily on the aftermath of abuse for children who have already suffered, but very little attention is given to the perpetrators of this abuse and how the root cause can be addressed to prevent a tragic life for the children.

These challenges underscore the critical importance of addressing maternal mental health and providing comprehensive support systems for mothers navigating the complexities of parenthood. This pilot study measures the impact of Drama Therapy Intervention (DTI) on family dynamics in Lebanon by targeting refugee women, who are impacted by compounded trauma leading to the maltreatment of their children.

### 2.2. Understanding Lebanon’s Refugee Population

Lebanon, a small country grappling with multiple, overlapping crises, has been described as a “failing state” (Alef, 2021). Hyperinflation, currency collapse, and a disintegrating healthcare system have driven multidimensional poverty to record highs, especially among refugees, who, per capita, form the largest refugee population in the world (Government of the United Kingdom, 2023). Lebanon has faced two major waves of displacement: over 100,000 Palestinians in 1948 (Suleiman, 2011) and more than one million Syrians since 2011 (Janmyr, 2016). As of 2024, refugees make up 25–30% of Lebanon’s population (BMZ, 2024).

Instability has long shaped Lebanon. Since independence from France in 1946 (Hakim, 2019), the country has been marred by internal conflict, including a 15-year civil war (1975–1990) that left over 100,000 dead (Abouzeid et al., 2021), and a destructive 34-day war with Israel in 2006 (Alagha, 2008). However, the crisis that began in 2019 has had even more far-reaching consequences. Anti-government protests led to the resignation of the prime minister (Al Jazeera, 2019), sparking a banking crisis, currency collapse, and hyperinflation. By 2021, poverty had doubled, reaching 82% (UNESCWA, 2021). The 2020 Beirut port explosion killed over 200 people and caused extensive damage (El Zahran et al., 2022).

More recently, the Israel–Gaza war triggered cross-border violence in southern Lebanon, displacing over 100,000 people and leading to thousands of deaths and significant economic losses (UNDP, 2024). As with most humanitarian crises, the most vulnerable—especially marginalized groups like refugees—suffer the most.

Refugees in Lebanon face persistent legal and structural barriers. Lebanon is not a signatory to the 1951 Refugee Convention and does not formally recognize refugees (Janmyr, 2016). Palestinian refugees have limited rights and legal status akin to seasonal workers (Al-Natour, 1997), while Syrian refugees face burdensome requirements for legal residency (UNHCR, 2015). Many remain undocumented due to poverty, legal obstacles, and a lack of viable pathways (Amnesty International, 2024). UNRWA and UNHCR oversee Palestinian and Syrian refugee affairs, but both agencies are overstretched and underfunded.

These systemic challenges compound the economic crisis. Refugee poverty rates exceed those of the host population (Kabbanji & Kabbanji, 2018), and school dropout rates are disproportionately high, particularly in camps (UNHCR, 2021). Access to healthcare, education, and utilities remains limited. Beyond material deprivation, refugees face lasting psychological and social consequences, including trauma from war and prolonged displacement.

### 2.3. Why Focus on Refugee Mothers?

Globally, refugees and internally displaced persons (IDPs) represent about 1% of the population, with 40% being children (UNHCR, 2019). Displacement exacerbates risk factors for child maltreatment, such as poverty, inadequate services, and social instability (LeBrun et al., 2015; World Health Organization, 2020). In Lebanon, these risks are magnified. The country reports the highest rates of child physical punishment in the Middle East (Usta et al., 2012), with 57–82% of children exposed to some form of maltreatment (El-Jardali et al., 2018). Refugee children are significantly more vulnerable, with higher rates of abuse than their Lebanese peers.

Refugee women, especially mothers, suffer from elevated mental health challenges due to war, displacement, and insecure living conditions. Studies show depression affects up to 31% of refugees, with women disproportionately impacted (Naal et al., 2021). PTSD is also prevalent, affecting 61% of this population (Haddad Kreidie et al., 2016). Poor parental mental health is strongly linked to child maltreatment and negative family dynamics (Lopes et al., 2021; Islam et al., 2022).

### 2.4. What Can Be Done?

Access to mental healthcare in Lebanon is limited by cost, stigma, and a lack of infrastructure (El Chammay, 2016), while child protection systems remain underdeveloped (El-Hoss, 2023). In this context, interventions addressing parental mental health and family well-being are urgently needed.

This study explores the potential of drama therapy to improve emotional regulation and family dynamics among refugee mothers. As a flexible, multi-modal Intervention, Drama Therapy has shown promise in supporting refugee women (Sakhi et al., 2022). Further research is needed to evaluate its effectiveness in reducing child maltreatment and informing relevant policy and practice.

## 3. Methodology

This pilot study was designed as a preliminary investigation into the feasibility, acceptability, and psychosocial impact of a Drama Therapy Intervention (DTI) on refugee mothers in Lebanon. It employed a partially mixed, sequential explanatory mixed-methods design in which quantitative data were collected and analyzed first, followed by qualitative data to help interpret the quantitative findings. This design was selected to evaluate both measurable outcomes and participants’ lived experiences, in line with GRAMMS (Good Reporting of a Mixed Methods Study) recommendations.

### 3.1. Study Objectives and Hypotheses

The primary objective was to assess changes in refugee mothers’ parent–child relationships and parental anger levels following 12 sessions of DTI. The secondary objective was to explore participants’ perceptions of family dynamics and emotional regulation, and to identify recommendations for future psychosocial interventions.

#### Hypotheses

**H0.**  *Refugee mothers will show no significant changes in their parent–child relationships or parental anger levels after 12 sessions of Drama Therapy Intervention (DTI).*

**H1.**  *Refugee mothers will show significant improvements in their parent–child relationships and reductions in parental anger levels after 12 DTI sessions.*

### 3.2. Methodology Design

The study followed a partially mixed, sequential explanatory design, wherein the quantitative phase was conducted first to capture measurable changes, and the qualitative phase followed to contextualize these results. Integration of findings occurred at the interpretation stage, where qualitative data helped to explain and deepen understanding of the quantitative outcomes.

This is a pilot feasibility study and was designed in alignment with the CONSORT 2010 extension for pilot and feasibility trials, with key features including clear objectives, rationale, and a discussion of sample size, intervention delivery, and limitations. This study is not a randomized controlled trial; however, we are in the process of pursuing prospective registration with the Lebanese Ministry of Health to enhance transparency.

The quantitative component used a repeated-measures design with two scales administered pre- and post-intervention: The Child–Parent Relationship Scale (Driscoll & Pianta, 2011) and the Parent Anger Scale (Gavita et al., 2011). The qualitative component consisted of focus group discussions (FGDs) to gain insight into participants’ parenting experiences and changes in family dynamics.

The intervention was implemented in collaboration with two NGOs: Makani in Beirut and Anamel in Bchamoun. Outreach, space provision, and participant recruitment were coordinated through these centers. The DTI program was designed and led by Farah Wardani, RDT, with study design by Dr. Lina H. Kreidie and research assistance from Suzanne Wehbe.

### 3.3. Study Population

The study enrolled 25 refugee mothers (22 completed the full intervention), recruited through two NGOs in Lebanon: Makani in Beirut and Anamel in Bchamoun. Inclusion criteria included refugee status (UNHCR/UNRWA registration), age 18+, basic literacy, and no recent participation in psychosocial programs. Most participants were aged 26–45, married (96%), unemployed (68%), and had three or fewer children. Educational attainment was generally low, with most having completed only elementary or secondary schooling.

Due to a lack of demographic data on refugee mothers in Lebanon, sample size determination was based on Jiang et al.’s (2023) meta-analysis of 25 drama-based intervention studies. A minimum of 20 participants was deemed sufficient for this pilot study.

### 3.4. Study Procedure

#### 3.4.1. Phase 1: Pre-Intervention Data Collection

Held in October 2023, this phase involved administering a demographics questionnaire and the two standardized scales. Sessions were conducted in person, and participation was voluntary and anonymized.

#### 3.4.2. Phase 2: Drama Therapy Intervention (DTI) Program Structure

The 12-week Drama Therapy Intervention (DTI) program was implemented in a group setting and followed Emunah’s five-phase model of drama therapy, with a thematic focus on motherhood, children, and family dynamics. While the inherently flexible nature of drama therapy allows for adaptability, all activities were intentionally grounded in core therapeutic concepts and tailored to address specific psychosocial challenges related to parenting and familial relationships. Techniques such as role-play, movement, storytelling, and improvisation were employed to foster emotional expression, increase self-awareness, and enhance interpersonal connection.

Each session opened and closed with ritualistic practices designed to establish and later dissolve the therapeutic “safe space.” The opening ritual involved breathing exercises to help participants connect mind and body, relieve accumulated stress, and prepare for emotional exploration. The closing ritual centered around a collaborative musical exercise: participants selected a familiar song, rehearsed it collectively, and performed it together—creating a shared moment of reflection and closure.

The first four sessions emphasized trust-building and group cohesion. Introductory activities such as “Name Tag” (sharing names and personal facts), “Walk in Space” (exploring spatial awareness), and “Roses and Thorns” (sharing weekly highs and lows) facilitated connection and emotional openness—essential prerequisites for deeper therapeutic work.

Sessions five through eight focused on emotional exploration and the internalized roles of motherhood. In “Dance Leader,” participants took turns leading expressive movement, linking physicality to maternal identity. Imaginative and reflective exercises like “Mirroring” and “Face in the Mirror” encouraged non-verbal communication, self-perception, and critical reflection on how past trauma, upbringing, and personal narratives shaped their current roles as caregivers. This phase deepened participants’ self-awareness and emotional insight, aligning with the program’s goal of enhancing maternal emotional clarity.

The final phase (sessions nine to twelve) addressed family relationships, personal growth, and parenting transformation. “Social Atom” helped participants visually map familial roles and emotional proximity, encouraging insight into relational dynamics. In “If I Weren’t, I Would Have Been,” participants imagined alternate life paths, fostering a sense of agency and hope. These sessions emphasized emotional intelligence, leadership, and child-centered parenting. Participants were encouraged to shift from control-based strategies to more attuned and responsive parenting styles.

By integrating emotional healing with skill-building, the DTI equipped refugee mothers with tools to not only improve family relationships but also to apply these changes within their communities—laying the foundation for sustained personal and interpersonal growth beyond the program.

#### 3.4.3. Phase 3: Post-Intervention Data Collection and FGDs

Post-intervention data were collected in March 2024 from participants who completed at least 8 sessions. FGDs were conducted in February 2024 to qualitatively assess program impact. Sessions were recorded, transcribed, and analyzed using phenomenological thematic analysis. Participants had a two-week window to withdraw their data post-collection.

### 3.5. Mixed Methods Integration

The rationale for using a mixed-methods approach was to allow quantitative findings to be supported, explained, and expanded upon through qualitative insights. Integration occurred during the interpretation phase through triangulation, where thematic narratives were used to corroborate or challenge quantitative trends. For example, observed reductions in parental anger were illuminated through participants’ descriptions of increased emotional awareness, self-care, coping strategies, and empathy.

### 3.6. Ethical Considerations

The study followed ethical guidelines approved by LAU’s IRB on 10 October 2023 RE: IRB #: LAU.SAS.LK1.10/Oct/2023 Protocol Title: Drama Therapy as a Tool for Peace and Conflict Resolution Dynamics in Family. Risks were minimal, limited to possible emotional discomfort due to trauma recall. These were mitigated by experienced facilitators and a referral system to mental health services. Informed consent, confidentiality, and the right to withdraw were emphasized. Data were anonymized and stored securely, with no identifying information collected.

### 3.7. Strengths and Limitations

This study offered a unique understanding of the experiences of mothers and the impact of DTI in regulating their parenting skills and mitigating anger. The study focused on a marginalized and often overlooked population in the larger landscape of research in the humanities and psychology. The mixed-methods approach provided a rounded understanding of both quantitative changes and the subjective experiences of the mothers who participated in the study. The questions tackled in this research (can parent–child relationships be improved, and can parents’ anger levels be regulated and reduced through a creative art therapy intervention?) offer areas for expansion and further research.

This study had certain limitations, starting with the sample size and lack of a control group. The sample size of 22 participants was well below the desired amount; however, logistical challenges limited the number of participants reached. The small sample size, however, offers a starting point for this kind of research. Secondly, we lacked a control group. The golden standard for research is the control group to ensure that the measured differences or changes are not purely due to environmental or social changes, or due to the passage of time. This study did not have a control group, once again due to logistical issues. The somewhat unstable conditions many refugees experience in Lebanon, ranging from unstable living conditions to precarious legal status, made it difficult to maintain a controlled research environment.

Despite these limitations, this study highlighted the possibilities for alternative forms of community-based interventions to tackle the complex topic of parenting. Future research should aim to continue to explore how mothers or parents can be supported and how psychosocial interventions can have an impact beyond the immediate beneficiary.

This study lays the groundwork for future fully powered, controlled trials with broader sampling and formal integration of MM reporting frameworks like GRAMMS.

## 4. Findings and Analysis

This study utilized a mixed-methods approach. The findings of the study are presented in two sections, the first being the quantitative findings, followed by the qualitative findings. In the first section, descriptive findings are presented in order to provide an overview of the population, followed by the scores of the pre- and post-intervention PAS and CPRS questionnaires. Finally, the qualitative findings are presented, allowing a more in-depth understanding of the impact of the program on the parenting relationship.

### 4.1. Quantitative Analysis

In Table 1, the PAS results show a significant reduction in both the experience and expression of anger after participating in the program, with the average experience score decreasing from pre-DTI 41.04% to post-DTI 23.92% and the expression score dropping from pre-DTI 21.64% to post-DTI 9.64%. This suggests that participation in the program significantly helped the mothers reduce their levels of anger and develop better emotional regulation skills.

Table 2 demonstrates notable changes in the CPRS. The closeness score saw a slight reduction from pre-DTI 43.44% to post-DTI 40.84%, indicating a reasonable shift as reflected in the substantial decrease in the conflict score from pre-DTI 36.72% to post-DTI 32.08%, suggesting that conflict between mothers and children was less frequent post-intervention. The mother–child emotional dependence fared a low but stable score (13.8 to 13.12) before and after the DTI sessions. Mothers feel the responsibility to express the compassion and love they feel when taking care of their children.

The findings suggest that the DTI helped mothers reduce their parental anger and improve their emotional responses toward their children, which subsequently led to reduced conflict in the relationship. The slight decrease in closeness might reflect healthier emotional boundaries, indicating that while the mothers have reduced conflict and better anger management, they may also be fostering a more balanced relationship that encourages emotional independence in their children. These results collectively support the hypothesis that drama therapy can positively influence family dynamics.

The quantitative results from the study align closely with the core concepts that the drama therapy programs were built around. The significant reduction in parental anger, as reflected by the PAS scores shown in Table 1, demonstrates the practice and expressive regulation of the parent–child relations reflecting the impact of the program in the mother’s emotional regulation, ability to navigate difficult conversations, and effectively meet their children’s needs. The notable decrease in conflict scores in Table 2 on the CPRS scale indicates that the program’s focus on child-centered parenting techniques, such as setting healthy boundaries and establishing routines, helped in improving mother–child relations and families getting along better. In addition, the minor reduction in closeness, coupled with stable emotional dependence scores, suggests a healthier balance between mothers and children. This could be potentially linked to the program’s focus on developmental stages and milestones, as well as fostering emotional independence in children. The program’s comprehensive approach, covering topics such as mother rage, self-care, and protective behaviors, seems to have played a role in enhancing mothers’ capacity to show positive behavior and emotional regulation, which has led to a more peaceful family dynamic.

### 4.2. FGD: Qualitative Analysis: Thematic Analysis

This section analyzes the narratives of 20 mothers who participated in two FGDs. These discussions took place in two different NGOs, one located in a refugee camp in the capital, Beirut, and the other in the suburb of Beirut, Bchamoun.

The purpose of this qualitative analysis is to examine the impact of the DTI on the mother’s emotions, social practices, and relational aspects with their children. This research highlights that refugee women experience compounded past and present traumas, which are reflected in their relationships with their children.

A common behavioral issue was evident in their verbal and non-verbal communication, which was staged during the DTI sessions. After building trust between participants in the first sessions, the middle four sessions of the program focused on emotional exploration and reflection on the roles of motherhood and personal identity. The analysis revealed how motherhood became central to their sense of self within the family’s social structure. The final sessions emphasized relational family dynamics, shifting toward fostering personal growth, developing leadership, and promoting child-centered parenting strategies. Visualizing and mapping their roles within the familial and social structure facilitated a deeper exploration of family roles and dynamics.

The FGD narratives reflected how the DTI sessions encouraged participants to discuss their feelings about their relationships and identify areas for growth and improvement. Despite continuing to experience stress and trauma mainly due to financial and living conditions, almost all mothers actively shared their thoughts during the discussions. However, the impact of compounded trauma and their inability to dedicate sufficient time and attention to their children often manifested as verbal and physical abuse.

In turn, the mothers emphasized how the DTI sessions helped them become more aware of their self-identity as mothers, active social units, and key players in family dynamics. As a result, they began shifting away from maltreating their children, giving them more attention, ceasing verbal and physical abuse, and ultimately fostering their children’s healthy development.

The qualitative analysis of the mothers’ narratives is based on the following themes.

#### 4.2.1. Parental Influence on Children’s Development: Role Modeling, Parenting Behaviors, and Evolving Approaches

The focus group discussions explored traditional parenting behaviors and techniques, including role modeling, alongside new, evolving approaches to parenting. The discussions examined how both conventional and contemporary methods influence children’s development, highlighting shifts in parental strategies and their impact on children’s behavior.

Gender role constructions emerged as a central theme in nearly all participants’ narratives. They described how their own upbringing shaped their parenting behaviors while acknowledging that the DTI sessions helped them realize that the mother–child relationship is not solely about disciplining children or assigning household chores but also about sharing learning experiences and making time for play. This realization was captured in one participant’s story:

Culture plays a significant role in shaping parenting practices. In some households, traditional values remain deeply ingrained. Our parents’ generation adhered to different beliefs and practices. I grew up in a household where girls were expected to wake up early and complete household chores, particularly when the mother was pregnant or giving birth. My mother gave birth to ten children and was often unwell, so I had responsibilities from a very young age. Looking back, I wonder how I managed it all.

Another participant reflected similarly:

by the time I was in first grade, I was already washing dishes. Now, when I observe my 9-year-old daughter, I am reminded of my own childhood. It is clear that our parents may not have fully understood their rights or ours as children. They should have recognized that we had the right to education, leisure, and the opportunity to enjoy childhood without being burdened by household duties. Childhood should be a time for exploration, learning, and play, not just mastering household chores. Unfortunately, this pattern was common in my neighborhood, where girls as young as 10 took on full household responsibilities and were often discouraged from pursuing education. Many were expected to marry by the age of 14.

Confirming the strong influence of traditional disciplinary actions, several participants shared how financial stressors made their parenting approaches even harsher, as echoed by one mother:

Unfortunately, my daughter does not attend school due to financial constraints. Nevertheless, I always encourage her to explore technical skills or pursue a profession, such as makeup artistry or crafting handmade goods. After DTI, I realized that teaching her household chores is the last thing on my mind. There are societal expectations that confine girls to domestic roles, perpetuating a cycle that limits their opportunities.

Another participant shared how the DTI sessions inspired her to prioritize quality time with her child: “The sessions also encouraged participants to prioritize quality time with their children.” One mother shared: “I now make a conscious effort to spend more quality time with my child, cherishing those moments together.”

In a more serious case, one mother described how stress and harsh disciplinary methods affected her daughter:

My eldest daughter is difficult to handle, unlike my youngest. She doesn’t comply! If she makes me angry, I pull her by her hair or ear. Then she went through a stressful phase” she was under pressure from both her father and me. She developed a urinary reflex and started unconsciously wetting herself. But after the sessions, I never raised a hand on her again. I always feel guilty afterward.

These accounts highlight the importance of parental role modeling. Children often mimic their parents’ behavior, making it essential for parents to embody the values they wish to instill. One mother reflected:

Raising a child is not just about telling them what to do; it is about practicing what you teach them, something I learned during the sessions. Now, I notice how my daughter behaves the same way, even when talking to me.

Another participant emphasized how the DTI helped her understand the value of apologizing to her children: “Before, I never thought to apologize to my child if I yelled at them. Now, when I realize I have hurt my child’s feelings, I say sorry. It makes her so happy and relieved.”

While some participants recognized the need for change, others found value in traditional parenting methods. One mother stated:

I never perceived my parent’s methods as wrong. On the contrary, as I matured, I came to realize they were right. We were raised in an environment without violence or hitting, and as a result, we raised children who respect us. My sister, who has 13 children, has raised them all well. She is sincere with her children, as are my other siblings with theirs. I feel that the only issue with the previous generation was having too many children. Because they gave birth to so many, each child did not receive enough attention.

However, the importance of giving children proper attention remains a crucial factor in their physical and mental well-being, as emphasized by the Child Abuse and Neglect (n.d.) Welfare Information Gateway (2022).

Most mothers expressed how DTI helped them adopt new parenting strategies that create a safe and stable environment for their children’s healthy development. One participant reflected on this transformation:

Initially, we followed our parent’s approach. However, after the sessions, we started employing different techniques with our children, considering each case carefully. The sessions also filled us with positive energy and enabled us to better understand children’s thoughts-even the youngest ones- and to respect that each child has their own perspective. It is the mother’s role to learn how to respond to them. Previously, we were taught that a baby only needs to be fed and cleaned. However, after the sessions, we realized that every gesture of a baby communicates something; they are trying to engage with us. We did not have this perspective before.

Another participant shared a similar sentiment:

My children are still young, but I have learned the importance of making them feel safe and comfortable sharing everything with me, including their concerns. Even though they are young, it is crucial to teach them. For instance, if my child does not like a certain meal, I do not force them to eat it. It is important to give them space.

One mother confirmed how the DTI also helped mothers manage their anger and handle stressful situations more effectively.

I benefited greatly from these sessions. I used to react harshly to my son because he was nervous, and I would respond with nervousness as well. However, since joining the sessions, I have learned how to manage my emotions, and he has started to respond positively.

Another mother echoed this change: “I was always angry, and I used to hit them. But now, I realize that my stress is not their fault, so I have stopped hitting them.”

Accordingly, the FGDs confirmed the study’s hypothesis: Refugee mothers experience significant changes in their relationships with their children and in their parental anger levels after attending 12 sessions of DTI. Through these sessions, mothers learned parenting strategies that improved their listening and communication skills, helped them control their anger, and encouraged them to allocate playtime with their children. As one mother passionately expressed:

Now, I believe in giving my children freedom. If my son wants to play, I let him play because, after all, he is still a kid. When he does something wrong, I prefer to explain things calmly rather than resorting to hitting or yelling. I rely on communication and role-playing to convey my point of view.

#### 4.2.2. Foundations of Stability and Peace in Family Life: A Sociological Perspective on Stability

One of the primary goals of DTI for refugee women is to equip mothers with the skills necessary to foster a more stable and healthy family dynamic. The sessions focused on enhancing communication, active listening, and clear expression, while also working to empower women’s emotional intelligence. The ultimate aim was for these skills to positively reflect in their homes and communities.

Building on this, participants shared personal reflections that highlight how stability, whether emotional, financial, or physical, contributes to a sense of peace within the family. One participant described peace as:

To me, peace is when my family and I feel safe, loved, and content. While problems are inevitable and often beyond our control, as long as we can live in a place we cherish and provide for ourselves financially, that is peace. Peace is not necessarily about the physical location or country we reside in because conflicts exist everywhere. It is about creating and sharing a sense of tranquility within our small family unit, knowing that our extended family members are safe and that we can always reach out to them to check on their well-being.”

Expanding on the idea of stability, a Syrian refugee participant linked stability to having a home rather than merely a house, emphasizing that true stability comes from providing care and nurturing for the entire family. She stated:

There is no stability. Due to inflation, we fear that we may be compelled to return to Syria at any moment. Honestly, I felt more stable in Syria than I do here. Here, stability feels partial. While it is true that we live in one house and do not frequently move, it is not our own house, so we do not experience true stability. I do not feel secure in this situation. To me, stability means residing in one home and nurturing it, but here, stability feels incomplete.

Similarly, another participant associated stability with being surrounded by family:

To me, stability means Syria. Even if I did not have a room or a house, I feel stable when I am with my mother, at my brother’s house, or even just seeing my neighbors. Being with my family is what brings me stability. I long for the day when I can go back.

These narratives highlight the idea that the nuclear family unit is a fundamental source of stability and peace. They also reinforce the importance of empowering trauma-impacted women, not only for the well-being of their families but also for fostering a more peaceful community. Considering the complexities and pivotal role of motherhood, the DTI’s emphasis on the mother’s role in family dynamics is essential in achieving both stability and peace.

#### 4.2.3. The Role and Complexities of Motherhood

Motherhood is about care, tenderness, and sacrifice, something almost all mothers agreed upon. It is a deep connection with one’s child, rooted in love and the desire to ensure their well-being. For many mothers, motherhood surpasses the daily acts of caregiving; it is an identity, a bond, and a lifelong responsibility. It involves not just nurturing a child physically, but also emotionally and spiritually. It is a journey that shapes a mother’s life, often redefining her sense of self as she navigates the challenges and rewards of raising a child. One participant noted:

Motherhood is tenderness. It is about providing children with care and tenderness and valuing their opinion. It involves being involved in everything the child experiences, ensuring they feel safe to share everything with you. This is the essence of motherhood. A mother should empathize with her children, understanding their words and feelings, and addressing their concerns.

While they recognized the joy of being mothers, many blamed situational burdens such as displacement and financial struggles for their inability to give their children the attention and resources they need. “Our kids argue about everything, and I cannot deal with this. I cannot handle their demands. I get tired just listening to them.” one mother shared. Another participant expressed the overwhelming responsibility of motherhood:

This can be exhausting for the mother. Considering each child’s mental well-being, psychological state, likes and dislikes, and experiences at school…the responsibility grows exponentially. Even with just four children, I already feel overwhelmed. I can’t imagine how challenging it must be for someone with 13 children.

While many mothers expressed feeling overwhelmed by the challenges they face, they also highlighted the importance of shared responsibility within the family unit. As much as mothers carry the caregiving burden, fathers, too, have vital roles to play. However, many mothers shared a sense of frustration with their husbands’ involvement, or lack thereof, in the day-to-day responsibilities of raising children. This lack of support, both emotional and practical, can further exacerbate the strain that mothers already feel in their roles.

Many mothers referred to fathers as the primary providers, responsible for meeting their children’s material needs: “When I am unable to meet my children’s needs, I tell them to go to their father, but he is always stressed and unable to do so. He ends up yelling at them a lot because he is overwhelmed.”

Another participant shared her perspective on the role of fathers:

I feel that my husband, and men in general, prioritize work and their external roles over domestic matters. They may not be as involved in raising children, which can be exhausting. When our son misbehaves in his presence, my husband often remains silent, seeming detached. It feels like he is in another world.

These revelations highlight how stressful situations contribute to dysfunctionality within family dynamics. The participants’ perspectives on their roles as mothers, their relationships with their children, and their interactions with their husbands affirm the importance of DTI in empowering mothers, hence, enhancing their personal growth, improving their ability to listen carefully, communicate effectively, and create emotional stability for their children.

#### 4.2.4. Impact of the Program on Personal Growth and Community Well-Being

The program was tailored to help mothers process past trauma, cope with stress, prioritize self-care, and navigate social relationships. This was reflected in the participants’ discussions on personal growth and community well-being. Almost all participants expressed how the DTI had significantly helped them, as one mother shared:

Before joining the program, I was struggling significantly. I realized that I tended to overthink many things, particularly when it came to feeling like my efforts at home were never enough. Memories of my life in Syria and everything I had lost would often trigger anger within me. However, through attending the sessions, I have learned to be grateful for the good health of myself and my family. I have also noticed a significant reduction in the intensity of my anger.

Another mother described how the program helped her develop techniques to cope with stress:

It has shifted our perspective on numerous aspects of life. While stress and problems may not disappear entirely, we have learned valuable techniques for coping with them effectively. Personally, I have gained so much from this experience. One of the highlights for me was discovering the benefits of physical activity, particularly walking.

Many mothers realized the importance of self-care in maintaining their mental and emotional well-being:

Through this program, I have learned the importance of giving myself even just a few minutes for self-care. I have realized that it is crucial not to let external stressors affect my health and well-being. I have also come to understand that it is perfectly okay to explore new avenues, especially when seeking psychological support, which is often considered taboo in our culture.

Another mother highlighted the importance of self-care in building resilience:

It is how we care for ourselves. We learned from these activities how to create space for ourselves. When you feel better, you have more energy to face any problem or challenge. I am more confident when dealing with others in my community. I encourage others who are suffering from trauma to undertake therapy, especially drama therapy that involves engaging in sports, venting, walking, sharing our feelings, and expressing ourselves. It is very beneficial, especially for us as new mothers.

The DTI techniques for managing daily and situational stressors not only helped women with self-care but also improved their relationships with their children and husbands:

Before the program, my mental state was terrible. I would constantly blame my husband for even the smallest things. However, things have improved since I joined the program. We now communicate better.

These positive changes were also reflected in their social interactions: “I have noticed a decrease in shyness and a significant increase in confidence. I now find myself engaging in conversations more readily than before.”

These testimonies illustrate how the DTI played a crucial role in fostering personal growth, strengthening family dynamics, and promoting emotional resilience within the community.

## 5. Conclusions

The impact of childhood maltreatment and the sensitive topics of family dynamics, especially in the context of populations who have historically faced many challenges, is an uncomfortable discussion. This study aimed to address the many challenges that arise when parenthood intersects with compounded trauma. In line with this, the use of DT provided a platform for the mothers to interact and build trust amongst themselves, use verbal and body language to express themselves, and share their challenging experiences in a group format. Twelve sessions of DT interventions helped the participants in coping with ongoing crises, mainly focusing on their own self-growth, improving their communication with their children, spending more time with them, and better listening to their needs. This reduction in child maltreatment is a significant lesson learned to psychologically empower women and help create peaceful family dynamics.

While the findings suggest promising directions for therapeutic intervention using DT in post-displacement contexts, the absence of a control group prevents any definitive claims about causality. Future studies should include a control group to establish comparative baseline data and isolate the effects of the intervention. Furthermore, this study sheds light on the need to do further research on the importance of mental health in rebuilding peace in zones of conflict.

## Figures and Tables

**Table 1:** Parent Anger Scale—Experience and Expression.

Parent Anger Scale—Experience and Expression		
	Pre	Post
PAS—Experience	41.04	23.92
PAS—Expression	21.64	9.64

**Table 2:** Parent–Child Relationship Scale.

Parent-Child Relationship Scale		
	Pre	Post
CPRS—Closeness	43.44	40.84
CPRS—Conflict	36.72	32.08
CPRS—Dependence	13.8	13.12

## Data Availability

The quantitative data and FDG transcripts used to support the findings of this study are available from the corresponding author upon request.

## Abbreviations

CPRS	Child–Parent Relationship Scale
DTI	Drama Therapy Intervention
FDG	Focus Group Discussion
IDP	Internally Displaced People
IRB	Institutional Review Board
LAU	Lebanese American University
PAS	Parental Anger Scale
